# Practicing the Same

**Published:** 1886-06

**Authors:** 


### PRACTICING "THE SAME."

Our alert contemporary, of the *Southern Clinic* tripped us up once on a *lapsus linguæ.* We now return the compliment:

In his "leader" in June, Brother Bryce gravely asks: Should the State grant license to a physician, who, after a fair trial, has demonstrated his unfitness to practice the same successfully?" Eh? Bro. Bryce? Well, Homer once nodded.

---

THE HOMŒOPATHS have petitioned Gov. Ireland to appoint a homœepath on the Board of Regents, and they also demand a division of "State patronage;" citing the fact that "an allopath" fills every State position where a medical man is required. How absurd to suppose a homœopath could fill a position where a medical man *is* required!!

---

"PROGRESS," is the name of a new medical journal to be issued at Louisville, in July, by Raymond & Co., and edited by Prof. Dudley S. Reynolds. We cordially welcome Dr. Reynolds back to the ranks of journalism. We have missed his fluent and flowery pen.

---

Dr, W. A. Morris, who contributes the paper on Differentiation of Croup and Diphtheria in this number, is a practitioner of fifty years experience, and his opinions and views are entitled to, and receive, from all who know him, much respect. Read the paper.

---

THE TEXAS DRUGGIST is the name of the latest venture in medical journalism in Texas. We have received Vol. 1. No. 1. It is to be a quarterly, and appears to be run in the interest of a large drug and grocery house in Waco. Dr. H. L. Taylor's name appears at the head of the editorial columns.

---

NATIONAL PATROL OF THE COAST BY REVENUE CUTTERS FOR THE PREVENTION OF INTRODUCTION OF CONTAGIOUS DISEASES.

Circulars have been issued from the United States Treasury Department to medical officers of the Marine Hospital Service, directing them to "cruise actively" with revenue cutters under their respective command upon the outer lines of their respective cruising grounds, and to exercise especial vigilance in inspecting all vessels arriving from foreign ports or from Southern ports of the United States," with the view of preventing the introduction into the United States of cholera and other infectious diseases. If an infected vessel, or one from an infected port is overhauled, she is to be sent to the nearest quarantine station. This is ordered by the President, to assist local authorities in the maintenance of quarantine against infectious diseases as provided by Sec. 4792 of Revised Statutes. Act of April 1878

